# A potential anatomic subtype of short bowel syndrome: a matched case-control study

**DOI:** 10.1186/s12876-016-0425-4

**Published:** 2016-01-29

**Authors:** Wencheng Kong, Jian Wang, Rongchao Ying, Yousheng Li, Huicheng Jin, Qi Mao, Danhua Yao, Mingxiao Guo

**Affiliations:** Department of Gastroenterological Surgery, Hangzhou First People’s Hospital, School of Clinical Medicine, Nanjing Medical University, Hangzhou, 310006 China; Intestinal Rehabilition and Transplant Center, Jinling Hospital, School of Medicine, Nanjing University, Nanjing, 210002 China; Department of Gastroenterological Surgery, Linyi People’s Hospital, Shandong, 276000 China; Department of Surgery, Jinling Hospital, School of Medicine, Nanjing University, 305 East Zhongshan Road, Nanjing, 210002 China

**Keywords:** Short bowel syndrome, PN weaning, Ileum, Bristol stool scale, Anatomic subtype

## Abstract

**Background:**

Fundamental researches suggest that ileum presents greater adaptive potential than the jejunum. However, few studies estimate the association between ileum and adaptive potential in human. To discover the association, we conducted this matched case-control study.

**Methods:**

A 1:2 pair-matched, case-control study was conducted from January 1, 2001 to January 1, 2015 in Intestinal Rehabilition and Transplant Center. The case group was ileum predominated (IP) group and the control group was jejunum predominated (JP) group. Demographic data, medical history and progression of each patient were collected.

**Results:**

There were 24 IP cases and 48 JP controls in this study. The cumulative probabilities of parenteral nutrition (PN) weaning in IP group were higher than that in JP group. The Bristol stool scale scores of IP group were lower than that of JP group at third month. The Cox proportional hazards regression model confirmed that IP had a higher odds of PN weaning (OR = 2.69; 95 % CI: 1.27, 5.70, *p* = 0.01) as compared with JP group. The conditional logistic regression with 1:2 matching also confirmed IP group had a higher odds (OR = 4.84; 95 % CI: 2.02, 11.56, *p* <0.01).

**Conclusions:**

Our results indicated that ileum presents greater adaptive potential than the jejunum in nutrition and fluid absorption. And a potential anatomic subtype of short bowel syndrome was proposed. Further research need to be conducted to more fully understand the adaptive potential of ileum besides nutrition and fluid absorption.

## Background

Short bowel syndrome (SBS) is a disabling malabsorptive disease usually following massive resection of the small intestine and characterized by the inability to maintain their nutritional status through normal oral intake alone [1]. For those irreversible intestinal failure patients, intestinal transplantation offers the potential to regain intestinal function and clinical nutritional autonomy. Along with improvements in immunosuppression, operative techniques and critical care, the survivals of patient and graft have gradually increased over time [2]. Whereas, it is still low as compared with the survival of patients who receive long-term parenteral nutrition [2, 3]. Recent years, some progress has been achieved in intestinal rehabilitation therapy, especially in the pharmacologic (glucagon-like peptide 2 [GLP-2] analogue and growth hormone) treatment which may augment the adaptive process and help patients wean off parenteral nutrition [4, 5]. Another important factor which effects intestinal adaptation is anatomic feature, including the length, anatomic site and function of residual intestine.

Interestingly, it seems that ileum presents greater adaptive potential than the jejunum [2, 6]. However, as far as we know, this conclusion is mainly concluded from animal study [7, 8]. Therefore, our understanding of intestinal adaptation still requires exploration in humans. And current study can only confirm the ileum decrease diarrhea and steatorrhea because of its absorption of water [9]. The other benefits, such as electrolyte balance and long transit time, are all in theory. Little clinical evidence is found in comparing the intestinal adaptation between ileum and jejunum.

Consequently, to further clarify the difference between ileum and jejunum in intestinal adaptation, we conducted this case-control study in our Intestinal Rehabilition and Transplant Center (IRTC).

## Methods

### Study design

The study was performed in accordance with the Declaration of Helsinki of the World Medical Association. With the approval of Institutional Review Committee of Jinling Hospital, we retrospectively reviewed all SBS patients between January 1, 2001 and January 1, 2013 in IRTC, China. SBS patients were identified by searching the diagnosis of “Short Bowel Syndrome” in the electronic medical record systems. Individual operative notes were physician-abstracted to obtain additional patient and operation information. A cohort of 356 SBS patients was achieved.

We elected two groups from the cohort and defined them as ileum predominated group (IP group, case group) and jejunum predominated group (JP group, control group). To be eligible as a case or control, the ileocecal valve and the colon were preserved in these patients. No surgery on the stomach, duodenum, or pancreas. The length of the intestine was between 100 and 20 cm. All patients must have longer than 2 years of adaptive phase and be followed. Intestine transplantation, malignancy, irradiation injury and Crohn’s disease were excluded. Benign diseases, such as mesenteric vascular disease and volvulus and trauma, were included. IP patients were defined that the ratio of residual ileum/jejunum was larger than 4:1. Inversely, the ratio of JP patients was less than 1:4. IP cases and JP controls were matched 1:2 randomly by sex and length of intestine(±10 cm). Patients who met the inclusion criteria were selected by two independent researchers.

### Content and data collection

The primary end-point of this study was development of intestinal adaptation, including weaning from parenteral nutrition (PN) and tolerance of enteral nutrition (EN) or regular diet during our study period (ending at January 1, 2015). The definition of “wean from PN” was absolute enteral independence and intermittent PN or fluid replacement was not included. We used Bristol stool form scale to score the typical stool form [10]. The secondary end-points were the hepatobiliary complications with PN management: steatosis, cholestasis, and gallbladder sludge/stones. We diagnosed “Steatosis” according to physical examination which might notice the liver was enlarged when the abdomen was palpated, blood tests including elevations of the serum alanine aminotransferase (ALT) and aspartate aminotransferase (AST) and ultrasonic diagnosis. Cholestasis was defined by elevation of conjugated bilirubin >2 mg/dL. Gallbladder sludge/stones were detected by ultrasound. Other chronic complications, such as metabolic bone disease and renal insufficiency, were also recorded.

**Fig. 1:**
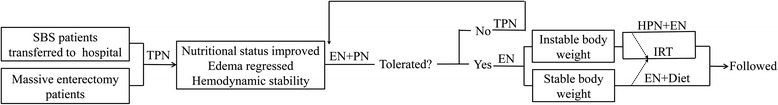
Schematic illustration of treatment procedure of short bowel syndrome and follow-up

Demographic, anatomic and therapeutic features of both group patients were collected. In our center, we had a procedure in dealing with SBS patients, which was illustrated in Fig. 1. If SBS patients from other medical centers were hospitalized, they would accept bowel rest and PN therapy until nutritional status improved and tissue edema regressed. If patients had undergone massive enterectomy in our center, they required PN for 7–14 days in order to maintain hemodynamic stability and pass the crisis. All patients were provided approximately 25–30 kcal/kg/day and 1.0–1.5 kg/day of protein. Patients would accept EN gradually via mouth, nasogastric tube, nasojejunal tube, percutaneous endoscopic gastrotomy tube or percutaneous endoscopic jejunostomy tube. EN started with amino acid formula (Vivonex, Nestle’, Minneapolis, USA) or peptide-based formula (Peptisorb, Nutricia, Wuxi, China), followed by a transition towards whole protein formula (Nutrison or Nutrison Fiber, Nutricia, Wuxi, China). The total calory and protein goal of EN combined PN were consistent with those of PN. If patients developed diarrhea, multiple antidiarrheal drugs includes antimotility agents, antisecretory drugs, antibiotics and probiotics were given to effectively control fecal output. Diarrhea was defined as having three or more loose or liquid stools per day, or as having more stools than is normal for that person. Once patients were EN intolerance mainly including high gastric residuals and vomiting, they would receive PN again. If tolerated, patients would receive home parenteral nutrition (HPN) and EN or intestinal rehabilitation therapy (IRT) according to the intestine function and patients’ wills. The IRT program was performed as described by Byrne et al. in 1995 [11]. If patients were EN tolerated but instable in body weight, they also underwent IRT. If patients were stable in body weight and EN tolerated, they were all encouraged to eat small and frequent meals. Some of them received IRT in order to wean EN and have normal diet. We routinely followed these patients through phone calls, and in-person visits with a well-designed questionnaire. This questionnaire main content included Bristol stool scale scores, starting and ending time of new nutrition method, BMI and complications. Each patient was followed at least four times at 1^st^, 3^rd^, 6^th^ and 12^th^ month. If patients had not weaned off PN in first year, they would be irregular followed-up.

### Statistical analysis

Cox proportional hazards regression and conditional logistic regression were used to estimate the odds of PN dependence stratified by jejunoileostomy types. Univariate analyses of demographic, anatomic and therapeutic features were performed to describe the study population. Missing data ranged from 0 to 5 % for all independent variables studied. Shapiro-Wilk W test for testing normality were conducted on all continuous variables. Normally distributed continuous variables were analyzed using Student’s t test and paired t-test. Categorical variables were analyzed using Chi-square test or Fisher’s exact test if expected frequency was less than five. The cumulative percentage of patients weaned over time was plotted using the Kaplane-Meier survivorship analysis and log-rank test for statistical comparison. Significance was set as *p* <0.05.

## Results

**Fig. 2:**
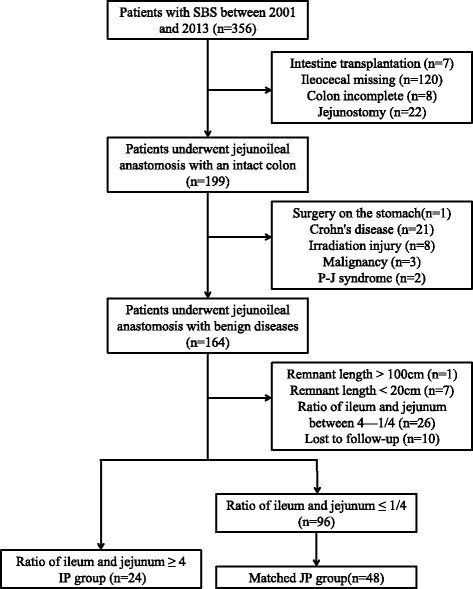
Flowchart of patient inclusion and exclusion. P-J syndrome, Peutz-Jeghers syndrome

Figure 2 illustrated the selection of patient. Eventually, 24 IP cases and 48 JP controls were selected. The demographic and clinical characteristics of both groups were summarized in Table 1. The characteristics of JP patients were similar to that of IP patients, but longer jejunum (49.5 ± 26.3 cm) and shorter ileum (8.4 ± 7.1 cm).

**Table 1:** Demographic and clinical characteristics of two groups

Characteristic	IP group (*n* = 24)	JP group (*n* = 48)	*p* value
Patient demographics			
Age (years),median (range)	40(26–63)	45(30–65)	0.42
Male gender (%)	67 %	67 %	1.00
BMI, kg/m^2^ (Mean ± SD)	17.5 ± 2.6	18.0 ± 2.5	0.35
Transferred patients	3	5	0.29
Etiology for SBS			0.80
Mesenteric vascular disease	13	23	
Small bowel volvulus	7	12	
Intestinal adhesion/obstruction	2	7	
Others	2	6	
Anatomic features			
Remnant intestine length,cm(Mean ± SD)	54.8 ± 28.8	57.9 ± 29.4	0.68
Jejunum, cm	7.3 ± 5.4	49.5 ± 26.3	**<0.01**
Ileum, cm	47.5 ± 25.0	8.4 ± 7.1	**<0.01**

Table 2 showed both groups had similar duration of PN or EN support, but the duration of PN+EN was significantly prolonged in JP group than that in IP group. About 1/3 (33 % in IP group, 42 % in JP group) of the patients accepted IRT. Almost all patients (88 % in IP group, 85 % in JP group) presented diarrhea after initiating EN. Steatosis and cholestasis were the other two main complications presented in roughly half of patients. The incidence of gallbladder sludge/stones in IP group was lower than that in JP group, but did not have a statistical significance. The other complications were rare during hospitalization.

**Table 2:** Therapic characteristics and complications of two groups

Characteristic	IP group (*n* = 24)	JP group (*n* = 48)	*p* value
Time of PN (Mean ± SD, d)	10.8 ± 2.8	10.7 ± 2.4	0.87
Time of PN+EN	7.5 ± 3.1	10.3 ± 3.7	**0.002**
Time of EN	4.4 ± 1.7	4.8 ± 2.0	0.42
Intestinal rehabilitation therapy	8(33 %)	20(42 %)	0.49
Complications in hospital ^a, b^			
Diarrhea	21(88 %)	41(85 %)	0.71
Catheter-related infections	2(8.3 %)	3(6.3 %)	0.34
Steatosis	11(46 %)	19(40 %)	0.61
Cholestasis	5(21 %)	12(25 %)	0.22
Gallbladder sludge/stones	1(4 %)	5(10 %)	0.26
Metabolic bone disease ^c^	2(8 %)	3(6 %)	0.34
Renal insufficiency	1(4 %)	1(2 %)	0.45
Complications during follow-up ^a^			
Death	1(4 %)	2(4 %)	0.45
Catheter-related infections	7(29 %)	16(33 %)	0.20
Steatosis	13(54 %)	23(48 %)	0.62
Cholestasis	8(33 %)	17(35 %)	0.86
Renal insufficiency	2(8 %)	5(10 %)	0.32
Nephrolithiasis ^d^	1(4 %)	6(13 %)	0.20
Gallbladder sludge/stones ^d^	1(4 %)	5(10 %)	0.26
Metabolic bone disease ^d^			
All cases	0(0 %)	2(4 %)	0.44
Tested cases	0/5(0 %)	2/8(25 %)	0.43

^a^ Some are overlapping

^b^ Occurred during hospitalization and diagnosed by the latest test results before discharge

^c^ Diagnosed by dual-energy X-ray absorptionmetry measurements of bone mineral density in suspect patients

^d^ Only a portion of patients were examined

**Fig. 3:**
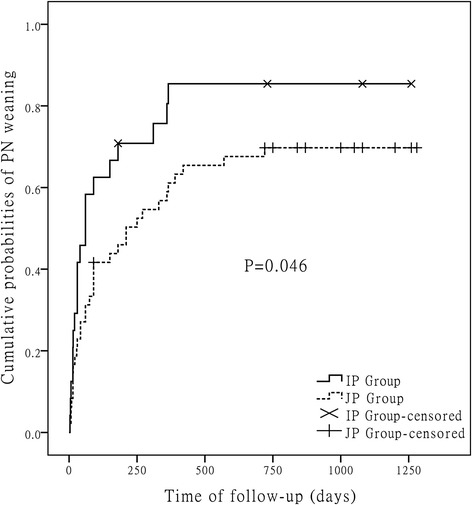
Cumulative probabilities of PN weaning in IP group and JP group during follow-up

**Fig. 4:**
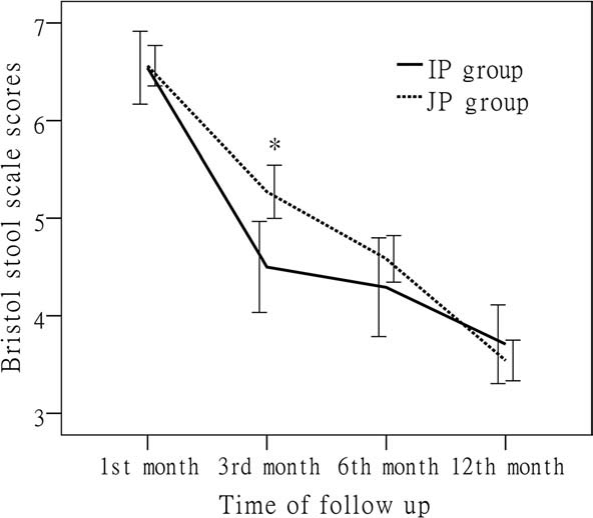
Bristol stool scale scores of IP group and JP group during follow-up. * *p* = 0.003

The average follow-up time was 968.5 ± 252 days. The cumulative probabilities of PN weaning were shown in Fig. 3. The curves of two groups had statistically significant difference (*P* = 0.046). The incidence of PN weaning in IP group (83.3 %) is higher than that in JP group (68.8 %). The changes of Bristol stool scale scores of two groups were shown in Fig. 4. The Bristol stool scale scores of both groups decreased to normal levels gradually during 1 year follow-up. However, the score of IP group (median, interquartile range; 4, 4–5) was significantly lower than that of JP group (5, 5–6, *p* = 0.004) at third month. The complications during follow-up were shown in Table 2. Three patients (1 IP patient, 2 JP patients) were dead during our study period. One IP patient and one JP patient were dead caused by end-stage liver disease and another JP patient were dead caused by infection (suspecting catheter-related infections). There were no statistical significances in complications during follow-up. But the complications of nephrolithiasis, gallbladder sludge/stones, and metabolic bone disease may be underestimated.

Table 3 showed adjusted odds ratio (OR) for PN weaning, calculated by Cox proportional hazards regression. Compared with JP group, IP had a significantly higher odds of PN weaning (OR = 2.69; 95 % CI: 1.27, 5.70, *p* = 0.01). The younger age (OR = 0.94; 95 % CI: 0.88, 1.00, *p* = 0.06) or higher BMI (OR = 1.41; 95 % CI: 0.98, 2.03, *p* = 0.06) might increase the possibility of PN weaning, but the difference was not statistically significant. Table 3 also showed the odds of PN weaning estimated using the 1:2 case (IP patients) to control (JP patients) matched design. And the IP group had a higher odds (OR = 4.84; 95 % CI: 2.02, 11.56, *p* <0.01).

**Table 3:** Estimated risks of PN weaning

	Unadjusted model			Adjusted model		
	Odds ratio	95 % CI	*p*-value	Odds ratio	95 % CI	*p*-value
a) Using Cox proportional hazards regression						
IP group^a^	1.74	[0.99, 3.05]	**0.05**	2.69	[1.27, 5.70]	**0.01**
Age	1.01	[0.99, 1.03]	0.59	0.94	[0.88, 1.00]	0.06
Male	0.84	[0.46, 1.54]	0.57	1.67	[0.68, 4.08]	0.26
BMI	1.06	[0.95, 1.18]	0.32	1.41	[0.98, 2.03]	0.06
Etiology for SBS^b^	1.14	[0.58, 2.42]	0.70	0.60	[0.28, 1.28]	0.18
Remnant intestine length	1.07	[1.05, 1.09]	**<0.01**	1.09	[1.06, 1.12]	**<0.01**
b) Using conditional logistic regression with 1:2 matching^c^						
IP group	4.84	[2.02, 11.56]	**<0.01**			

^a^ IP group, ileum predominated group; Jejunum predominated group was reference group

^b^ Mesenteric vascular disease was covariates; Small bowel volvulus was reference group

^c^ IP cases and JP controls were matched by sex and length of intestine (±10 cm)

## Discussion

As far as we know, this was the first clinical study to address the question whether the ileum have a better functional adaption than the jejunum. The functional adaption included transporter/cell, crypt cell, transit time, nutrition and fluid absorption [6]. In fact, we had more interest in nutrition and fluid absorption. Our study suggested that the ileum predominated SBS patients were easier to wean PN than jejunum predominated SBS patients within 1.5 years following resection. This result was in agreement with previous animal study but with a shorter duration of intestinal adaptation. It was generally believed that most of intestinal adaptation in adults occurs within 2 years following resection [12]. And some studies suggested the adaptation can occur beyond 2 years especially in adults. A study which followed 124 SBS patients who were weaned off PN showed more than a quarter of them were weaned off PN beyond 2 years following resection [13]. So we inferred this type of SBS defined as type 3 anatomy in some studies had a faster intestinal adaptation because the intact colon had a good adaptation in nutrition and fluid absorption [14].

Diarrhea was another main difficulty affecting SBS patients’ quality of life. Massive intestinal resection, gastric hypersecretion, intestinal bacterial overgrowth, bile acids malabsorption and other factors contributed to diarrhea in SBS patients [15]. In this study, the scores of Bristol stool scale of patients were improved continuously to normal value within 1 years following resection. It seemed that these patients’ diarrhea was improved more quickly as compared to other studies [16, 17]. But as compared between groups, IP patients had better scores of Bristol stool scale than JP patients at the third month. This result implied ileum might adapt faster than jejunum in controlling diarrhea. The ileum’s capability of bile acids absorption and potentiality of fluid absorption might be the mechanisms. While, the scores of Bristol stool scale had no significant differences between the two groups after 1 year adaptation. Besides, we found that the improvement of diarrhea always occurs earlier than weaning PN. This was a remarkable phenomenon which researchers should pay more attention to when study the intestinal adaptation.

Hormone therapy became a popular method to help SBS patients accelerate intestinal adaptation with successful application of growth hormone and GLP-2 [18]. Some studies showed intestinal hormones had multiple physiological effects in SBS patients because of lacking intestinal hormones. For instance, secretin and cholecystokinin played regulatory roles in hepatobiliary system which was often disorder in SBS patients [19, 20]. Serotonin and glucose-dependent insulinotropic peptide had a great effect on maintaining bone mass [21, 22]. As is well known, ileum and jejunum have different preferable hormones [18]. So we intended to find the differences of complications between IP group and JP group in this study. But no significant difference was found in duration of hospital stay or follow-up.

**Fig. 5:**
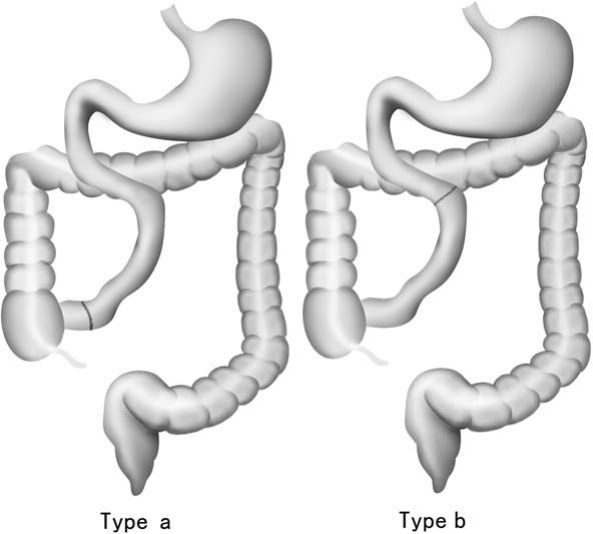
Intestinal anatomic subtype of short bowel syndrome: *Type 3a*, the main remnant intestine is jejunum; *Type 3b*, the main remnant intestine is ileum. The black line shows the anastomosis

In the regression analysis, we found IP group was associated with 2- to 5-fold greater probability of PN weaning than JP group. Remnant intestine length was another significantly associated factor. Besides, BMI and age were two potential factors in borderline. These two factors were supported by previous studies among SBS patients [3, 23]. In this study, we studied the difference between ileum and jejunum in patients’ intestinal adaptation. The existing evidence and indications suggested ileum has greater adaptive potential than the jejunum in nutrition and fluid absorption. Whereas, it still need prospective studies to demonstrate ileum’s adaptive potential in other aspects such as hormone and transit time. According to this study, we proposed to divide type 3 anatomy SBS into three subtypes: Type 3a, the main remnant intestine was jejunum; Type 3b, the main remnant intestine was ileum (Fig. 5); Type 3c, the mixed type.

There were several limitations of this article. This was a retrospective cohort study with no standardization protocol for patients to decide if and when to accept IRT or wean PN, thus introducing potential bias from unmeasured confounders.

## Conclusions

In conclusion, we found that the length and type (ileum or jejunum) were associated with an increased possibility of PN weaning in SBS patients. Our results indicated that ileum presents greater adaptive potential than the jejunum in nutrition and fluid absorption. Further research need to be conducted to more fully understand the adaptive potential of ileum besides nutrition and fluid absorption.

## Abbreviations

EN: enteral nutrition
IP: ileum predominated
IRT: intestinal rehabilitation therapy
JP: jejunum predominated
PN: parenteral nutrition
SBS: short bowel syndrome

## References

[1] O’Keefe SJ, Buchman AL, Fishbein TM, Jeejeebhoy KN, Jeppesen PB, Shaffer J (2006). Short bowel syndrome and intestinal failure: consensus definitions and overview. Clin Gastroenterol Hepatol.

[2] Thompson JS, Rochling FA, Weseman RA, Mercer DF (2012). Current management of short bowel syndrome. Curr Probl Surg.

[3] Vantini I, Benini L, Bonfante F, Talamini G, Sembenini C, Chiarioni G (2004). Survival rate and prognostic factors in patients with intestinal failure. Dig Liver Dis.

[4] Matarese LE (2013). Nutrition and fluid optimization for patients with short bowel syndrome. JPEN J Parenter Enteral Nutr.

[5] Jeppesen PB, Pertkiewicz M, Messing B, Iyer K, Seidner DL, O’Keefe SJ (2012). Teduglutide reduces need for parenteral support among patients with short bowel syndrome with intestinal failure. Gastroenterology.

[6] Tappenden KA (2014). Intestinal adaptation following resection. JPEN J Parenter Enteral Nutr.

[7] Appleton GV, Bristol JB, Williamson RC (1987). Proximal enterectomy provides a stronger systemic stimulus to intestinal adaptation than distal enterectomy. Gut.

[8] Thompson JS, Quigley EM, Adrian TE (1999). Factors affecting outcome following proximal and distal intestinal resection in the dog: an examination of the relative roles of mucosal adaptation, motility, luminal factors, and enteric peptides. Dig Dis Sci.

[9] Cosnes J, Gendre JP, Le Quintrec Y (1978). Role of the ileocecal valve and site of intestinal resection in malabsorption after extensive small bowel resection. Digestion.

[10] Lewis SJ, Heaton KW (1997). Stool form scale as a useful guide to intestinal transit time. Scand J Gastroenterol.

[11] Byrne TA, Persinger RL, Young LS, Ziegler TR, Wilmore DW (1995). A new treatment for patients with short-bowel syndrome. Growth hormone, glutamine, and a modified diet. Ann Surg.

[12] Buchman AL, Scolapio J, Fryer J (2003). AGA technical review on short bowel syndrome and intestinal transplantation. Gastroenterology.

[13] Amiot A, Messing B, Corcos O, Panis Y, Joly F (2013). Determinants of home parenteral nutrition dependence and survival of 268 patients with non-malignant short bowel syndrome. Clin Nutr.

[14] Carbonnel F, Cosnes J, Chevret S, Beaugerie L, Ngo Y, Malafosse M (1996). The role of anatomic factors in nutritional autonomy after extensive small bowel resection. JPEN J Parenter Enteral Nutr.

[15] Kumpf VJ (2014). Pharmacologic management of diarrhea in patients with short bowel syndrome. JPEN J Parenter Enteral Nutr.

[16] Cosnes J, Carbonnel F, Beaugerie L (1994). Functional adaptation after extensive small bowel resection in humans. Eur J Gastroenterol Hepatol.

[17] Mitchell JE, Breuer RI, Zuckerman L, Berlin J, Schilli R, Dunn JK (1980). The colon influences ileal resection diarrhea. Dig Dis Sci.

[18] Tappenden KA (2014). Pathophysiology of short bowel syndrome: considerations of resected and residual anatomy. JPEN J Parenter Enteral Nutr.

[19] Kanno N, LeSage G, Glaser S, Alpini G (2001). Regulation of cholangiocyte bicarbonate secretion. Am J Physiol Gastrointest Liver Physiol.

[20] Chandra R, Liddle RA (2007). Cholecystokinin. Curr Opin Endocrinol Diabetes Obes.

[21] Cui Y, Niziolek PJ, MacDonald BT, Zylstra CR, Alenina N, Robinson DR (2011). Lrp5 functions in bone to regulate bone mass. Nat Med.

[22] Mieczkowska A, Irwin N, Flatt PR, Chappard D, Mabilleau G (2013). Glucose-dependent insulinotropic polypeptide (GIP) receptor deletion leads to reduced bone strength and quality. Bone.

[23] Thompson JS, Weseman RA, Rochling FA, Grant WJ, Botha JF, Langnas AN (2014). Pre-resection gastric bypass reduces post-resection body mass index but not liver disease in short bowel syndrome. Am J Surg.

